# Survey of the *Solidago canadensis* L. Morphological Traits and Essential Oil Production: Aboveground Biomass Growth and Abundance of the Invasive Goldenrod Appears to Be Reciprocally Enhanced within the Invaded Stands

**DOI:** 10.3390/plants11040535

**Published:** 2022-02-17

**Authors:** Beáta Baranová, Eva Troščáková-Kerpčárová, Daniela Gruľová

**Affiliations:** Department of Ecology, Faculty of Humanities and Natural Sciences, Prešov University in Prešov, 081 16 Prešov, Slovakia; evatroscakova@gmail.com (E.T.-K.); daniela.grulova@unipo.sk (D.G.)

**Keywords:** Canadian goldenrod, morphological traits, essential oil productivity, management

## Abstract

Canadian goldenrod is one of the most widespread invasive neophytes in Europe with proven ecological and environmental consequences for the invaded plots. The morphological traits and productive features survey can offer a better insight view into the *S. canadensis* population ecology and the dynamic of its aboveground biomass growth. Equally, it can serve as a foundation for a balanced management proposal, with the aim of keeping an acceptable degree of Canadian goldenrod invasion. In the study, 600 specimens, collected at various phenological phases, from the twelve sampling stands in the eastern Slovakia, were processed. The obtained data were related to the degree of invasion, pH, soil moisture, overall stand area, and measure of interventions. Plants from the stands with a mild degree of goldenrod invasion (<50%), lower pH, and higher stand area were significantly lower and lighter; had a significantly lower number and weight of leaves; significantly shorter and lighter stems, in comparison to the plants from the stands with a heavy degree of invasion (>50%); a higher pH; and a smaller area. These plants also showed smaller essential oil productivity rate, and they achieved the growth peak a significantly later. Conversely, as the stand area decreased, and the *S. canadensis* % representation and soil reaction increased, goldenrods became significantly taller and heavier, with a higher number of leaves and a higher essential oil productivity rate. Canadian goldenrod shows, somewhat, a cyclical, *self-growth-reinforcing* *feedback*: the consecutive increase of the goldenrod’s aboveground biomass leads to an increase of its relative % abundance within the invaded stands. Consequently, the increase of the goldenrod’s relative % abundance leads to the plants aboveground biomass consecutive growth, and so on.

## 1. Introduction

Perennial herb *Solidago canadensis* L. (Asteraceae: Astereae), originating in North America, was brought and introduced into middle Europe as an ornamental, schizanthus, and melliferous plant in the middle of the 18th century. In the end, it unintentionally spread from the gardens to the natural environment. Today, Canadian goldenrod is one of the most widespread invasive neophytes in central and eastern Europe. Its distribution within the territory of Slovakia has considerably grown since the collapse of socialism in 1989, when the land changed from being state-owned to a privately owned. Thus, many pieces of lands remained without a known owner. The previously cultivated land became abandoned and lacked regular interference, such as mowing. Consequently, strewed spots of dense mono-specific goldenrod coenoses of different sizes are typical for the urban zones and agricultural landscapes, at present [1,2,3,4].

Although it is still being studied, *S. canadensis*, as an invasive neophyte, displays some superior features, which make it more successful in comparison to autochthonous species, and several possible mechanisms of its success have been described:

- the production of a high number of small, light-winged seeds, spreading mostly by the air, germinating rapidly in the high percentage and, with a wide tolerance for the different values of soil reaction, salinity, and moisture;
- robust asexual reproductive ability of the underground parts (rhizomes, nodes, stem bases), hereby just a naturalized population has a great capacity for a clonal growth, since the clonality is in general concurrently found to offer advantages that facilitate invasion [5];
- ability to occupy various types of habitats, including overloaded areas and those polluted by heavy metals [6,7]; and
- release of the allelopathic compounds, including essential oil (EO), which negatively affect seeds’ germination and the growth of the native species [8,9,10,11,12].

The success and persistence of *S. canadensis* have also been connected to various morphological traits: (a) contribution of Canadian goldenrod plants’ height to its relative % abundance (degree of invasion) within the invaded plots was pinpointed [13]; (b) the relationship between communities of soil arbuscular mycorrhizal fungi (AMF) and *S. canadensis* aboveground and belowground phenotypic traits as the plant’s height, number of leaves, chlorophyll content of leaves, rhizomes’ number, and roots’ biomass allocation was described [14,15]; (c) polyploidy of *S. canadensis* was connected with larger root systems [16]; and (d) significant influence of selected soil properties on *Solidago* height and inflorescence size was detected, too [17]. Equally, the plants’ quantitative productivity of essential oil, as the plant secondary metabolite, is nearly correlated with the plants’ growth and morphological traits and, indirectly, with the environmental variables [18,19]. Accordingly, a measure of the goldenrod biomass growth directly reflects the success of this invasive neophyte on the local scale and quasi predicts the future course of the invasion. Equally, the morphological features survey gives a basis for balanced and optimized management of *Solidago* and, subsequently, feedback on the effectiveness of various management practices applied with the aim of reducing goldenrod biomass or restoring the invaded plots [20,21,22].

*Solidago canadensis* concurrently displays several biologically valuable features—its biomass contains proteins, lipids, saccharides, vitamin C, carotenoids, and amino acids, which indicates that it could be a source of valuable products for the bioeconomy [23]. As the major compounds of its essential oil, monoterpenes, such as α-pinene, β-pinene, bornyl acetate, camphene, limonene and thymol, terpene β-elemene, and sesquiterepenes, such as β- gurjunene, δ-cadinene, germacrene D, and longifolene, were determined [24,25,26]. These components are used as the constituents of phytotherapeutic drugs for the chronic diseases treatment and account for antioxidant, antimicrobial, and antifungal activity [27,28]. These constituents are equally found to be responsible for goldenrod EO repellency [29], although the Canadian goldenrod EO insecticidal activity remains less studied [26]. *Solidago canadensis* EO was also confirmed to account for phytotoxicity [10,30]. The leaf extracts of the Canadian goldenrod were declared to have promising potential in the green synthesis of triangular and hexagonal gold nanoparticles (AuNPs) used in medicine [31].

On the other hand, the ecological and environmental impacts of the changes connected with goldenrod invasion are still not absolutely clear, nor are they unequivocal. Nevertheless, goldenrod biomass and the litter production should be, undoubtedly, reduced as much as possible. Although the effect of the *S. canadensis* invasion on the impacted ecosystems is still not absolutely clear, and nor is it unequivocal, the necessity of its removal is undoubtable, and even compulsory, according to actual Slovakian legislation.

The main goal of our study was to: survey selected morphological traits and the essential oil productivity rate of the invasive neophyte *Solidago canadensis* from the stands with the various goldenrod relative % abundances; describe the Canadian goldenrod aboveground biomass growth and EO yield at the different phenological phases; specifically assess distinctions in the *Solidago* morphological features and EO productivity in relation to various degrees of invasion and the selected variables used for the sampling stands characterization.

## 2. Results

### 2.1. Morphological Traits

Plant material processed for this study was collected during the growing season of 2014, from 12 sampling stands in the eastern Slovakia. Stands were proportionally divided into the three groups, with categories according to different degrees of *S. canadensis* invasion:

A category—including sampling stands A1–4 with a heavy degree of invasion, i.e., 75–100 Canadian goldenrod relative % abundance within the vegetation cover;

B category—including sampling stands B5–8 with a middle degree of invasion, i.e., 50–75 Canadian goldenrod relative % abundance within the vegetation cover; and

C category—including sampling stands C9–12 with a mild degree of invasion, i.e., 25–50 Canadian goldenrod relative % abundance within the vegetation cover.

Ten single plants (ramets) were collected from each of the sampling stands, overall, five times, i.e., at 5 sampling terms, which corresponded to various *S. canadensis* phenological phases: green goldenrods without inflorescence; goldenrods in full blooming; and goldenrods after active blooming period. Summarily, 600 specimens were processed for this study. Except for the morphological traits, the EO productivity rate was also determined.

Mean values and standard deviations (±SD) of the selected morphological traits (those determined on the, *fresh*, plant material before drying out), as well as EO yields, are listed in Table 1, Table 2 and Table 3. The differences between the sampling stands categories in the selected morphological traits and EO yields, assessed using One-way ANOVA, with three levels of significance (*p* < 0.05; *p* < 0.01; *p* < 0.001), are indicated in Table 1, Table 2 and Table 3 too.

#### 2.1.1. Morphological Traits—Different Phenological Phases

The mean *S. canadensis* plant’s height and the entire plant’s weight increased significantly from the 1st (May/June) to the 3rd (August) sampling term, when the highest and the heaviest plants were noticed. Subsequently, height and weight were balanced between the 4th (September) and 5th (October) sampling terms.

The stems were lowest in the 1st (May/June) sampling term and were significantly shorter in comparison to 2nd, 3rd, and 4th sampling terms. The highest stem weight was noticed during the first three sampling terms (May–August). In the late growing season, i.e., September and October, the stem’s weight decreased and was significantly lower in comparison to May–August.

The lowest number of all the leaves was observed at the 1st (May/June) sampling term, while the highest was at the 2nd (June/July) sampling term. Consequently, the number of all leaves decreased, till, in September and October, this decrease was significant (2nd June/July versus 4th September *p* < 0.05; 2nd June/July versus 5th October *p* < 0.001; 3rd August versus 5th October *p* < 0.01). The weight of the all leaves was the highest in the 2nd (June/July) sampling term. Consequently, no distinctions were observed between the separate sampling terms. However, the average weight of the single leaf was significantly higher at first, in comparison to other of the sampling terms. The ratio between the entire plant’s weight and all leaves’ weight was more than 3.5, in general, as well as in every category of sampling stands. The lowest number of the assimilating leaves was noticed in May. Consequently, their number significantly increased from June to August, and repeatedly significantly decreased in October. A similar pattern was observed concerning the weight of assimilating, green, leaves; however, the distinctions between the particular sampling terms were not significant. In opposition, the number, as well as the weight, of non-assimilating, brown, leaves were the highest just at the end of the growing season. Since the average weight of the single assimilating leaf decreased, those of the non-assimilating increased from the beginning to the end of the growing season. The mutual ratio of the assimilating and non-assimilating leaves changes on behalf of the non-assimilating leaves as the growing season came to the end.

Fully developed inflorescences were noticed in the 3rd sampling term in the first half of August. In general, neither the length nor the weight of the inflorescence changed significantly during the monitored period.

The highest relative water content of the entire plant, stem, and all the leaves was noticed in the 1st (May/June) sampling term and in inflorescence at the beginning of the blooming period. Consequently, relative water contents decreased, and the lowest were noticed in October.

#### 2.1.2. Morphological Traits—Different Degree of Invasion

**Figure 1:**
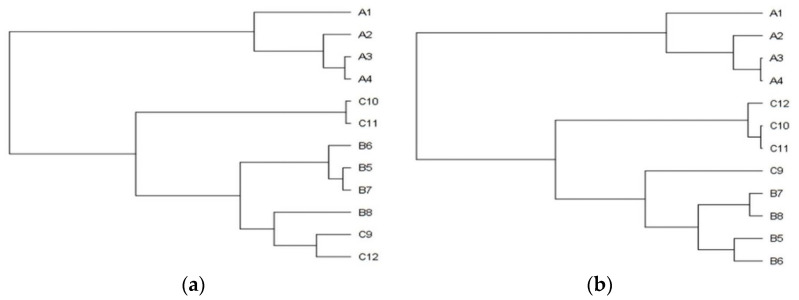
Dendrograms of the sampling stands classification according to *Solidago canadensis* (**a**) height and (**b**) entire plant’s weight. Stands with the different degree of invasion were classified using Cluster analysis, Ward’s method. Abbreviation and notes: A1–4 = heavily invaded stands with 75–100%, B5–8 = medial invaded stands with 50–75%, C9–12 = mildly invaded stands with 25–50% relative abundance of *Solidago canadensis* within the vegetation cover.

In general, the plants from the stands with the mild degree of invasion (25–50%) were significantly lower (A versus C *p* < 0.001; B versus C *p* < 0.01) and lighter (A versus C *p* < 0.05). The differences between the sampling stands in the plant’s height and the entire plant’s weight are also shown in the dendrograms (Figure 1a,b), where clusters, consisting of stands with the identical degree of invasion (A1–4, B5–8, C9–12), are clearly recognizable. The separated position of the group of stands with a heavy degree of invasion (A category) is obvious.

The plants with the longest stems were noticed within the stands with 75–100% *S. canadensis* relative abundance. The stem’s average length significantly decreased simultaneously with the decrease of invasion degree (A versus C *p* < 0.001; B versus C *p* < 0.001). The stem weight was, in general, significantly lowest in the plants from the C category of sampling stands (A versus C *p* < 0.001; B versus C *p* < 0.001).

The lowest number of all the leaves was observed in the plants from the mildly invaded stands (25–50% *S. canadensis* representation; A versus C *p* < 0.05; B versus C *p* < 0.001). Plants from the stands with mild degree of invasion had significantly lower weight of all leaves (A versus C *p* < 0.01; B versus C *p* < 0.01), as well as the single leaf’s weight, in comparison to plants from the A category (A versus C *p* < 0.01).

In addition, we did not notice the distinctions in the number of assimilating, *green*, leaves between the A, B, and C categories. However, assimilating leaves from the plants of mildly invaded stands showed significantly lower weight (A versus C *p* < 0.01; B versus C *p* < 0.05). Non-assimilating, brown, leaves from the plants of the C category showed a significantly lower count, as well as weight, in comparison to those of A and B categories (number: A versus C *p* < 0.05; B versus C *p* < 0.01; weight: A versus C *p* < 0.01; B versus C *p* < 0.05).

The plants from the heavily invaded stands had the longest and heaviest inflorescences. However, neither the length nor the weight was significantly different, according to the degree of the invasion.

Generally, no distinctions in the entire plant’s nor stem’s relative water contents were observed between the A, B, and C sampling stands categories. Only all the leaves and inflorescences from the plants of the heavily invaded stands had a significantly higher relative water content in comparison to those from the mildly invaded stands (leaves A versus C *p* < 0.05; inflorescences A versus C *p* < 0.05).

#### 2.1.3. Relative Daily Growth Rate

**Figure 2:**
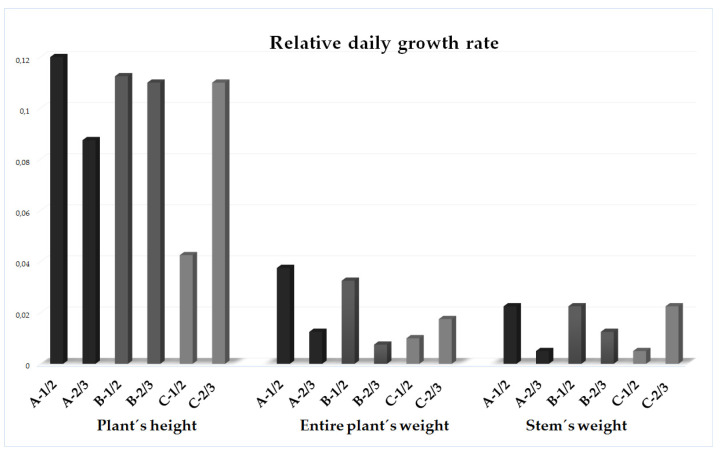
Differences in the relative daily growth rate of the selected morphological traits in relation to degree of *Solidago canadensis* invasion. Abbreviation and notes: A = heavily invaded stands with 75–100%, B = medial invaded stands with 50–75%, C = mildly invaded stands with 25–50% relative abundance of *Solidago canadensis* within the vegetation cover; ½—between 1st/2nd sampling terms (May–beginning of July), 2/3—between 2nd/3rd sampling terms (end of June and the first half of August).

Relative daily growth rate concerning selected morphological traits was, in general, the highest in the plants from the heavily invaded stands. The highest growth rate of the plant’s height, the entire plant’s weight, and the stem’s weight was noticed in the plants of the A and B categories between 1st/2nd sampling terms (May–-beginning of July). However, in comparison, the plants from the stands with just the mild degree of invasion (25–50%) showed their highest plant’s height, the entire plant’s weight, and stem’s weight daily growth rates to be belated, i.e., between the end of June and the first half of August (2nd/3rd sampling terms) (Figure 2). Belated daily growth rate was also noticed in the inflorescence, when, in plants of the A and B categories, flower weight relative daily growth was the highest between August and September, while, between September and October, it was highest concerning C category plants.

The average daily growth rate of all the leaves’ weight was the highest between May and the beginning of July (1st/2nd sampling terms) and from side of the degree of invasion.

### 2.2. Essential Oil Productivity Rate

Mean values and standard deviations (+SD) of the EO yields are listed in Table 1, Table 2 and Table 3.

**Figure 3:**
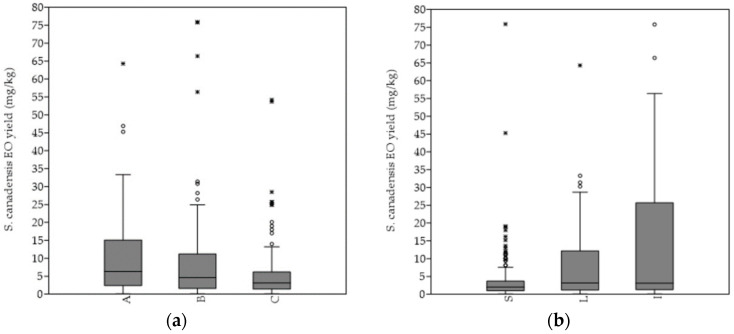
Differences in the essential oil productivity rate according to different *Solidago canadensis* (**a**) degree of invasion and (**b**) plant organs. Abbreviations and notes: A = sampling stands’ category with 75–100%, B = 50–75% and C = 25–50% relative abundance of *Solidago canadensis* within the vegetation cover; S—stems, L—leaves, I—inflorescences. Boxplot outliers are shown as * and °.

The hydro-distillation of the *S. canadensis* dry plant material yielded pale yellow oil. In general, the mean EO yield increased continuously till the 4th sampling term (September), when it was the highest, and significantly higher in comparison with every of the other sampling terms. A similar pattern was observed by evaluating the EOs’ yields from the separated plant organs, i.e., stems, leaves, and inflorescences, as well as according to degree of invasion. Generally, the highest average EO yield was obtained from the plants of the heavily invaded stands, while the lowest was obtained from those of the mildly invaded stands (Figure 3a). The mean EO yield obtained from the plants of the A category was significantly higher in comparison to those of the C category (*p* < 0.01). The highest EOs’ yields were, in general, extracted from the inflorescences, followed by leaves and stems (Figure 3b). The inflorescences’ mean EO yield was, in comparison, also significantly higher (I versus L *p* < 0.001; I versus L *p* < 0.001). The same pattern was equally observed within the separated A, B, and C categories.

### 2.3. Correlations

The plant’s height and overall plant’s weight positively and significantly (*p* < 0.05) correlated with all other morphological traits. The exception concerned number of all leaves and relative water content of the entire plant. The number of all leaves positively and significantly (*p* < 0.05) correlated with the stem’s length and weight. Length and weight of the inflorescence positively and significantly correlated with the stem’s weight. The entire plant’s relative water content did not correlate with any morphological traits evaluated.

The stems’ and leaves’ EO yields were mutually correlated with each other.

The stems’ EO yield was negatively and significantly correlated with the entire plant’s relative water content (*p* < 0.05). The leaves’ EO yield positively and significantly (*p* < 0.05) correlated with the stem’s length.

Categories of sampling stands did not mutually differ in the variables used for their characterization. However, we observed a significant positive correlation (*p* < 0.01) between the degree of invasion and soil reaction. Average pH was the highest within the stands with the highest *S. canadensis* % representation. On the other side, the significant negative correlation (*p* < 0.05) between the goldenrod relative % abundance and the overall area of sampling stand was detected. The stands with the lowest area had the highest goldenrod coverage, and vice versa.

**Figure 4:**
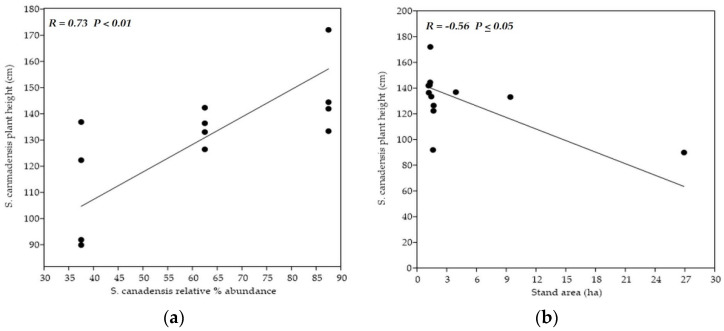
Simple linear regression between *Solidago canadensis* plant height and two variables. Models display simple linear regression between (**a**) plant height and the degree of invasion and between (**b**) plant height and overall area of sampling stands.

**Figure 5:**
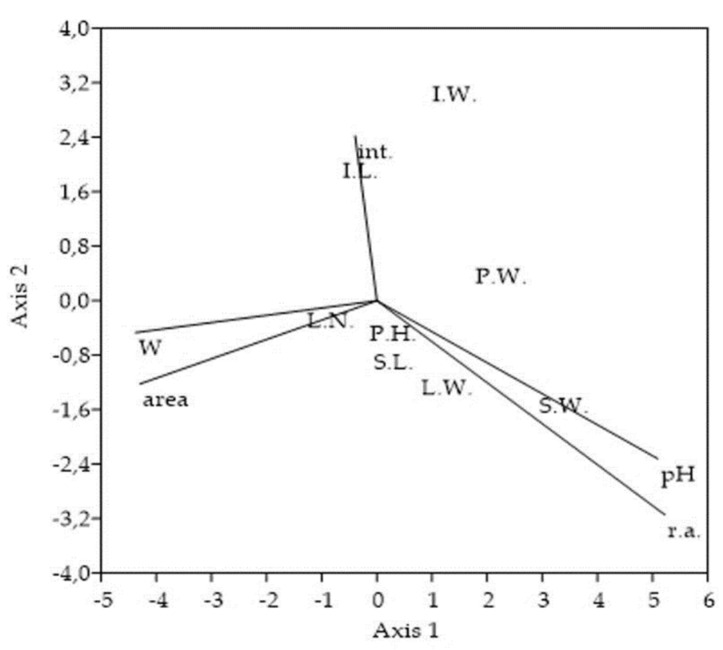
Ordination plot of the selected *Solidago canadensis* morphological traits and variables used for the sampling stands characterization. CCA ordination triplot shows association of 8 morphological traits to degree of invasion, soil reaction, soil moisture, stand area, and measure of interventions. The cumulative percentage variance of morphological traits-variables relation explained on Axis 1 = 70.26% and on Axis 2 = 20.92%, with eigenvalues for Axis 1 = 0.003 and for Axis 2 = 0.001. Abbreviations and notes: P.H.-plant’s height, P.W.-entire plant’s weight, S.L.-stem’s length, S.W.-stem’s weight, L.N.-all numbers of leaves, L.W.-all leaves weight, I.L.-inflorescence’s length, I.W.-inflorescence’s weight, r.a.-*Solidago canadensis* relative % abundance within vegetation cover, pH-soil reaction, W-soil moisture, area-overall area of sampling stands, int.-measure of interventions (number of mowings).

Concerning relations of morphological traits and EO productivity rate to variables used for the sampling stands characterization, plant’s height, stem’s length, and stem’s weight, as well as inflorescence’s humidity, were significantly (*p* < 0.01) and positively correlated with relative % abundance of *S. canadensis* within the vegetation cover, i.e., degree of invasion (Figure 4a). CCA analysis confirmed this ascertainment (Figure 5). The same pattern was observed concerning the soil reaction. The tallest plants, with the longest and heaviest stems, were noticed within the stands with the highest pH, which, concurrently, means those with the highest goldenrod abundance. Conversely, the aforementioned morphological traits significantly and negatively (*p* < 0.05) correlated with the overall stand’s area (Figure 4b). Only the leave’s weight was correlated with soil humidity, and none of the evaluated morphological traits was correlated with the measure of interventions, i.e., number of mowings.

Stem and leaf EO yields positively and significantly correlated with the soil reaction (*p* < 0.05). Moreover, leaf EO yield was positively correlated with the *S. canadensis* relative % abundance.

## 3. Discussion

Based on the results obtained, *S. canadensis* morphological features and essential oil productivity rate varied significantly during the growing season (Table 1, Table 2 and Table 3). In accordance with the other findings [7,20], we noticed the growth peak of Canadian goldenrod within the period of August and early September (Table 1, Table 2 and Table 3).

Moreover, goldenrod aboveground biomass growth significantly changed in the dependence on the invasion degree, soil reaction, and area of sampling stand. Summarily, sampling stands with the higher area were associated with only a mild degree of invasion (25–50%). Corresponding goldenrods were significantly lower and lighter, had significantly shorter and lighter stems, and had significantly lower numbers and weights of leaves (Table 3, Figure 4a,b). Equally, they achieved the growth peak significantly later in comparison to the plants from the stands with the middle and heavy degree of invasion (>50% r.a.) (Figure 2). Conversely, the stands with the lower area were associated with the middle to high degree of invasion (>50% r.a.). Simultaneously, as the *S. canadensis* relative % abundance increased within the vegetation cover, goldenrods became higher, heavier, and with higher numbers of leaves (Table 1, Figure 4a,b). Detected correlations, as well as differences between the stands, with various degrees of invasion, indicate a strong positive association between the *S. canadensis* aboveground biomass growth and its relative % abundance within the invaded plots (Figure 4a and Figure 5). Canadian goldenrod obviously prospers more as its relative % abundance within the invaded plot is higher, since the plants’ height and ramets’ biomass directly reflect the quality of habitat, where the invader grows [5].

Besides, the increase of the soil reaction simultaneously with the goldenrods density was observed too. However, the direct correlation is disputable because the impact of the *Solidago* invasion on the soil properties is very unambiguous [32].

Based on our results, Canadian goldenrod shows, somewhat, a cyclical, *self-grown-reinforcing feedback*, which could be explained as follows: consecutive increase of the goldenrod’s aboveground biomass leads to increase of its relative % abundance within the invaded stands. Consequently, the increase of the goldenrod’s relative % abundance leads to plants’ aboveground biomass consecutive growth, and so on.

The suggested above is consistent with the conclusion that the *Solidago* plants’ height is strongly related to its relative abundance, and, consequently, goldenrods’ height is, thus, directly responsible for the invasion success [13]. Our suggestion can provide understanding on the progressing increase of the number of ramets, as well as the clones of Canadian goldenrod, which were observed during the consecutive three years by the mapping of *Solidago* invaded plots with the help of various geospatial technologies. Moreover, the highest increase of both ramets and clones was observed just within the plots with the highest goldenrod % representation [33].

We supposed that the suggested phenomenon of *self-grown-reinforcing feedback* is based on several, mutually-associated mechanisms as described below:

Whereby the degree of invasion is higher and the goldenrods are taller, the shadowing within the invaded stands is more intense. Thereby the single goldenrod specimen’s height increases, as the competitiveness for the sunlight acquisition is one of the driving force of the *S. canadensis* growth [13]. This can explain why the tallest goldenrods were observed just within the plots with the highest *Solidago* coverage (Table 1, Figure 4a). Besides, taller goldenrods also induce more intensive shadowing to the native species, which supports competitive suppression [34], and leads to higher goldenrods success. However the taller and heavier goldenrods produce more biomass and litter, with higher N, P, and K contents [35]. Consequently, its faster decomposition rate leads to higher pool and availability of nutrients, which positively affects growth advantages of the Canadian goldenrod itself [17,36,37]. The enhanced nutrient cycling rate can equally accelerate development of the AMF communities within the invaded stands [38,39], as well as increase the soil microbial biomass (SMB) [35,37,40]. Conversely, AMF supports the increase of goldenrods below ground and total biomass, the plant height, number of leaves number, leaf chlorophyll content, number of rhizomes, or biomass allocation of roots [14,15]. SMB, through positive feedback on the soil processes, consequently, supports goldenrods biomass growth [35,37]. We suppose, that the biomass and litter positive contribution to *self-grown-reinforcing feedback* is intensified because the vast majority of the *Solidago*-invaded plots are without regular mowing. Thus, most of goldenrods biomass remains in the stand, available to be utilized, in comparison to native, regularly mowed grasslands, where the harvested biomass is removed. Furthermore, remaining goldenrod biomass and litter can release the macerate of the allelopathic secondary chemical compounds [41]. Yet, the various extracts from goldenrods rhizomes, stems, or leaves can inhibit germination, as well as radical elongation, of the native species [8,9,11,12,42]. The harmful impact on the autochthonous plants within the invaded plots supports competitive suppression, and herewith goldenrod *self-grown-reinforcing feedback*. The effect is supposed to accelerate as the plants are taller and heavier and, thus, the amount of plant biomass and litter increase.

Given the aforementioned, we suppose that the taller and heavier goldenrods: contain a more robust and dense root system working as the physical barrier, which possibly prevents rooting and growing the native species; requires more intense water and nutrient uptake, which leads to hardship of autochthonous vegetation; and creates more intense shadowing leading to higher soil moisture, which is the favorable condition for *Solidago* itself.

Absence of the regular mechanisms against goldenrod within the autochthonous plots, plus *hands-free* management, bring benefits, too, as the soil culturing and mowing decreased the growth of *S. canadensis* [7,20,43,44].

We also suppose that the intensity of *self-grown-reinforcing feedback* simultaneously increases with the goldenrod density within the stand and is likely more *spatially concentrated* within the stands with the smaller area.

The peak of the essential oil productivity rate was generally achieved within the period of August and early September (Table 1, Table 2 and Table 3).

The smallest plants from the plots with just mild degree of invasion (<50% r.a.) showed the lowest essential oil productivity rate (Table 3, Figure 3a). However, although the highest EO yields were obtained only from the tallest and heaviest plants of the heavily invaded stands (>50% r.a.) (Table 1, Figure 3a), essential oil productivity rate seemed to be relatively independent on the evaluated morphological traits. Nevertheless, a positive correlation between EO yield and morphological traits, such as, for example, plant height, is usually observed [18,19]. On the other hand, the amount of the essential oil produced by the *S. canadensis* stems and leaves showed association with soil reaction, as well as the degree of invasion, whereas the impact of the *Solidago* relative abundance on the inflorescence EO yield seems to only be indirect [18,19].

Despite very limited and variable data, *Solidago* essential oil seems to contain anti-germinative and phytotoxic potential [10,26]. Our other, processed, but yet unpublished, data indicate that Canadian goldenrod EO phytotoxicity varies not only during the growing season but also according to *Solidago* degree of invasion within the stand of the plants’ material origin. Except volatilization from the green plants, releasing of these secondary metabolites from the remained goldenrod biomass and litter can be supposed [41]. The harmful impact of the *Solidago* essential oil on the native plants germination and growth should be taken into account [10,26]. Suggested competitive suppression would increase simultaneously with the plants’ biomass, plus the degree of *Solidago* invasion, and with the decline of autochthonous flora [45].

### Management Proposal

Although the effect of the *S. canadensis* invasion on the impacted ecosystems is still not absolutely clear, and neither it is unequivocal, the necessity of its removal is undoubtable, and even compulsory, according to actual Slovakian legislation. In connection with our findings, reduction of both aboveground and underground biomass seems to be crucial when aiming to decrease the invasion degree or to keep it on a stable, not-growing level, since nothing else but mowing is found to be the most effective tool. A management proposal should be based on the very personalized and addressed access, according to the status of the single stand or area, which should be managed, in the manner perceptive to the quality of surrounding habitats and landscape structure [46].

Because of that, we hardly take into account, however, a bit of the controversial, but possible, benefits: in a fragmented, biologically depleted agricultural landscape, dominated by large-scale intensively managed sections of arable land and grasslands, which is quite typical for the territory of Slovakia and most post-communistic countries, in the context of lack or total absence of non-crop or native habitats, small-scale invaded plots could function as potential local refuges [47]. In addition, this could also act as a valuable additional source of pollen and nectar for a wide spectrum of insects before the winter period because of goldenrods blooming within the later part of the growing season in comparison to autochthonous species [48,49]. Just because of regular management of the surrounding predominated habitats, we presume that the probability of neophytes unregulated extension is very low [50,51].

On this basis, our management suggestions are as follows:

- invaded stands within the protected, vulnerable, and natural value areas, or those needing to be restored, should receive intensive management, including mowing two times per year, plus shallow ploughing/rotary tilling [21], applied, at the latest, when the inflorescences are already yellow, but before the full opening of the petals [22]; and
- invaded stands within the intensively managed agricultural landscape, without a special natural value, could receive extensive management, including irregular mowing, or mowing once per year [7,20], applied, at the earliest, when inflorescences are yellow gold and the petals begin to open [22].

Consequently, as the Canadian goldenrod concurrently contains several biologically valuable features, practical utilization of the harvested aboveground biomass should be subsequent as much as possible, to give the mowing management more than a superficial purpose. In the context of the management goal, removing of the harvested plants seems to be crucial, as the remaining *Solidago* biomass supports its growth via positive feedback on the soil processes [36], as well as through releasing the macerate with allelopathic effect [41] (as mentioned above).

## 4. Materials and Methods

### 4.1. Sampling Stands

**Figure 6:**
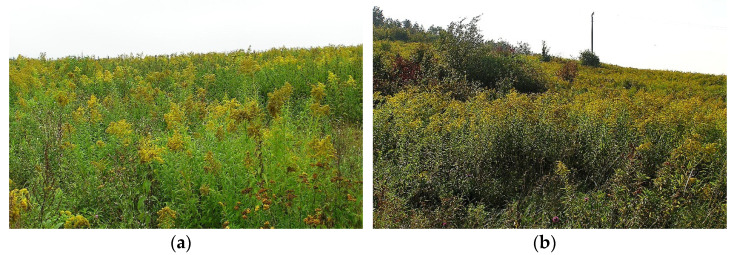
Examples of the *Solidago canadensis* sampling stands (**a**) number 3 and (**b**) number 4 (see Table 4) within the urban and suburban zone of the town of Prešov and surrounding villages in eastern Slovakia.

Material of the Canadian goldenrod (*Solidago canadensis* L., Asteraceae) was collected at twelve sampling stands localized within the urban and suburban zone of the town of Prešov and surrounding villages, in eastern Slovakia (Figure 6a,b). Sampling stands represent anthropogenic biotopes, previously used as the grasslands or arable lands (according to the Slovak national Register of Soil), and today being abandoned and without the regular interferences; however, the absolute accurate time period, which they are under in the invasion mode, is unknown. The stands mutually differed in the relative % abundance of the Canadian goldenrod. Species identification was provided according to Reference [1].

Each sampling stand was characterized by the following variables: (i) degree of Canadian goldenrod invasion, (ii) soil reaction, (iii) soil moisture, (iv) area of sampling stand, and (v) measure of interventions (Table 4).

Degree of invasion was defined on the base of relative % abundance (r.a.) of *S. canadensis* within the sampling stands’ vegetation cover and on the basis of the Braun-Blanquet cover-abundance scale [44]. Relative % abundance was visually estimated in the center of each sampling stand, at the time of full blooming, on the basis of three categories that were defined as follows: A—heavy degree of invasion = 75–100%, B—middle degree of invasion = 50–75%, and C—mild degree of invasion = 25–50% of the Canadian goldenrod % representation within the vegetation cover, which correspond 5th, 4th, and 3rd categories of the Braun-Blanquet cover-abundance scale, respectively. Each stand’s category was then represented by four replications.

To establish soil reaction and soil moisture, mixed soil samples were taken at every sampling term, for, overall, fivefold per research period. Samples consisted of three sub-samples randomly taken within the sampling plot (mentioned below), from the depth of 5–15 cm. Soil reaction (pH) was determined in a 0.01 mol·L^−1^ CaCl_2_ solution using WTW inoLAB ^®^pH 720 Laboratory Meter (Burladingen, Germany). Soil moisture was determined gravimetrically [52]. Mean values from the five measurements were determined using univariate statistic in statistical software PAST 2.17c [53]. Stand area in hectares (ha) was estimated according to the satellite pictures, whereby the border of the invaded stand could be clearly distinguished. Although most of the chosen stands were without regular mowing during the sampling period, we noticed a cutting process, which was then expressed as the measure of interventions, i.e., number of mowings.

From the pedological point of view, stands of the A category were characterized with the presence of slightly skeletal chernozem, brownsoil, and cambisols; stands of the B category were characterized with the non-skeletal luvisol, cambisol, and regosols; and stands of the C category were characterized with the medial skeletal cambisols and chernozem. The soils were middle to very heavy. Because of their relatively near mutual position, we supposed that the stands were under very similar climatic conditions.

### 4.2. Material Collection and Processing

Plants of Canadian goldenrod were collected during the growing season of 2014, within the research period from May to October, at five sampling terms, coresponding to various plants’ phenological phases:

- 1st sampling term—between 24 May and 9 June;
- 2nd sampling term—between 27 June and 13 July—the first two sampling terms were characterized by green plants, without inflorescences;
- 3rd sampling term—between 3 and 18 August—the third sampling term was characterized with fully developed inflorescences;
- 4th sampling term—between 3 and 23 September—goldenrod blossoming was coming to the end; however, inflorescences were still considerably yellow; and
- 5th sampling term—between 7 and 28 October—plants passed active blooming period.

Plant material was collected as near as possible to the middle of sampling stand, within the rectangular shape sampling plot of approximately 25 × 40 m. As the Canadian goldenrod forms clonal clusters of shoots (ramets), one ramet (shoot), from overall ten clusters, was then collected—clusters were randomly selected within the sampling plot, positioned at least 10 m from each other. In total, ten single plants (ramets) were collected from every sampling stand, and, at every of five sampling terms, summarily, 600 specimens were processed for this study. The following morphological traits were determined: (1) height of the entire plant (before the blooming period, including the also the group of small leaves on the plant apex, in the blooming period including also the inflorescence) in centimeters (cm), rounded off to one decimal place, determined directly in the field using telescopic flexible aluminum measure tape. Consequently, plants were cut on the bottom, nearest to the soil surface as possible, using garden shears. Then, plants were brought into the lab and processed immediately to evaluate selected morphological traits in the *fresh* condition, i.e., before drying out. Weights, in grams (g), were determined using a standard lab weighing-machine, and lengths, in centimeters (cm), were determined using telescopic flexible aluminum measure tape. All values were rounded off to one decimal place. After (2) weight of the entire plant was determined, leaves and inflorescence (in blooming period) were manually separated from the stem, and the following traits were determined: (3) number of assimilating, green, the group of small leaves on the plant apex was counted as the one leaf; (4) overall weight of assimilating, green, leaves; (5) number of non-assimilating, brown, leaves; (6) overall weight of non-assimilating, brown, leaves; (7) length of the stem; (8) weight of the stem; and (9) length of the inflorescence; (10) weight of the inflorescence. Then, separated stems, leaves, and inflorescences were left to freely dry by constant room temperature and in the dark for a 7-day period. The following traits were directly determined using the dried plant material: (11) weight of the dried stem; (12) overall weight of the dried assimilating, green, leaves; (13) overall weight of the dried, non-assimilating, brown, leaves; and (14) weight of the dried inflorescence. On the basis of the obtained results, we counted: (15) number of all leaves, as the sum of the assimilating, green, and non-assimilating, brown, all numbers of leaves; (16) weight of the all leaves in fresh condition; (17) weight of the all leaves after drying out, as the sum of the weights of assimilating, green, and non-assimilating, brown, leaves; (18) average weight of the single assimilating, green, leaf in the fresh condition; (19) average weight of the single assimilating, green, leaf after drying out; (20) average weight of the single non-assimilating, brown, leaf in the fresh condition; (21) average weight of the single non-assimilating, brown, leaf after drying out, where average weight of the single leaf was determined as the ratio of the leaves’ overall weight and the leaves’ number; and (22) weight of the entire plant after drying out, as the sum of the dried stem, all leaves, and inflorescence weights. Relative water content (%) was counted for (23) stem; (24) all leaves; (25) inflorescence; and (26) entire plant using following formula:

(1) $$\left(\frac{\left(\mathrm{weight}\;\mathrm{in}\;\mathrm{the},\;fresh\;condition,-\mathrm{weight}\;\mathrm{after}\;\mathrm{drying}\;\mathrm{out}\;\right)}{\mathrm{weight}\;\mathrm{in}\;\mathrm{the},fresh\;condition,}\right)\times 100.$$

On the basis of the distinctions in the values of selected morphological traits between the consecutive sampling terms, we determined (27) relative daily growth rate for plant height; (28) relative daily growth rate for plant weight; (29) relative daily growth rate for stem weight; (30) relative daily growth rate for all leaves’ weight; and (31) relative daily growth rate for inflorescence weight, using following formula:

(2) $$\frac{\left(\frac{\mathrm{sum}\;\mathrm{value}\;\mathrm{of}\;\mathrm{trait}\;\mathrm{from}\;\mathrm{consecutive}\;\mathrm{sampling}\;\mathrm{term}}{\mathrm{number}\;\mathrm{of}\;\mathrm{samples}\;}-\frac{\mathrm{sum}\;\mathrm{value}\;\mathrm{of}\;\mathrm{trait}\;\mathrm{from}\;\mathrm{previous}\;\mathrm{sampling}\;\mathrm{term}}{\mathrm{number}\;\mathrm{of}\;\mathrm{samples}}\right)}{\mathrm{number}\;\mathrm{of}\;\mathrm{days}\;\mathrm{between}\;\mathrm{sampling}\;\mathrm{terms}\;}.$$

### 4.3. Essential Oil Productivity Rate Assessment

Dried material of the stems, leaves, and inflorescences, coming from the ten goldenrod individuals, collected at identical sampling terms and sampling stands, were pooled according to previously determined weight of the entire, non-dried plant (in descending order), to obtain proportional weighted subsamples. Consequently, twenty grams of each subsample was weighed, ground in a blender, and hydro-distilled in a Clevenger-type apparatus for 2 h to extract essential oil. For the purpose of this study, the essential oil productivity rate was assessed through the EO yield, determined in milligrams per 0.020 kg of dry biomass (mg/kg). Practically, two to three subsamples per sampling stand/sampling term/plant organ were obtained and, the weight data concerning a total of 351 EO samples were used in the study to assess the Canadian goldenrod EO productivity rate.

### 4.4. Data Analysis

Minimal, maximal, and mean values and standard deviation (+SD) were determined using Univariate statistics. General differences between the sampling stands categories (A, B, according to degree of invasion, i.e., relative % abundance of the Canadian goldenrod) and between the sampling terms (1st–5th) in the selected morphological traits and yields of essential oil were assessed using Two-way ANOVA, with three levels of significance (*p* < 0.05; *p* < 0.01; *p* < 0.001). Differences between the separated sampling terms (1st–5th) within and between the sampling stands categories (A, B, and C) were assessed using One-way ANOVA, with three levels of significance (*p* < 0.05; *p* < 0.01; *p* < 0.001). Differences in the variables used for the stands characterization were determined, too. To depict observed distinctions, Descriptive Statistics were used. Selected morphological traits were mutually clustered using Cluster analysis and Ward’s method. Spearman’s Rs correlation test was used to assess possible correlations between the morphological traits/EO yields and variables used for the stands characterization, between morphological traits and EO yields, and morphological traits between each other. Simple linear regression analyze was used to depict observed significant correlations. The effect and significance of variables used for the stands characterization on the selected morphological traits and EO yields were examined using canonical correspondence CCA analysis in PAST, and data were log+1 transformed prior to analysis. All statistical analyses were performed using PAST, version 2.17 [53].

## 5. Conclusions

Results obtained within this study indicate a strong positive association between the *S. canadensis* aboveground biomass growth and the degree of its invasion. Canadian goldenrod obviously prospers more as its coverage within the invaded plot is higher. We suppose that *S. canadensis*, through various mechanisms, affects the habitats of its occurrence, thus making them more favorable for its growth.

Summarily, *S. canadensis* growth and its abundance within the invaded stands appears to be reciprocally enhanced: increasing goldenrods’ aboveground biomass enhances the consecutive increase of the *Solidago* invasion, and, conversely, increase of the Canadian goldenrod abundance is the driving force of its aboveground biomass increase, and so on.

Our detailed survey of the *S. canadensis* morphological traits and productive features, studied at various phenological phases and in relation to different variables used for the sampling stands characterization, offers a better insight view into the Canadian goldenrod population ecology and the dynamic of its aboveground biomass growth; contributes to the enlightenment of *S. canadensis* success and increase of its invasion degree at the local scale; and can be helpful for the balanced and optimized management proposal, which focuses on the keeping an acceptable degree of Canadian goldenrod invasion through a combination of mowing with the consistent practical utilization of the obtained biomass, including essential oil extraction.

## Figures and Tables

**Table 1:** Selected morphological traits and essential oil yields (mg/kg) of the *Solidago canadensis* from heavily invaded stands and significant mutual distinctions between the A versus B (º) and A versus C (*) categories of sampling stands, evaluated generally and for separated sampling terms, with three levels of significance (º, * *p* < 0.05; ** *p* < 0.01; *** *p* < 0.001).

Morphological Traits		A Category 75–100% (Heavy Degree of Invasion)				
		May/June	June/July	August	September	October
Plant height (cm)	***	100.5 ± 14.5	139.8 ± 14.9 *	167.8 ± 20.2 *	167.5 ± 22.3	159.4 ± 30.9
Entire plant weight (g)	*	35.2 ± 10.1 *	47.8 ± 16.3	55.1 ± 17.1	67.3 ± 34.9	62.5 ± 22.2
Relative water content of entire plant (%)		75.6 ± 6.6	61.7 ± 3.6	58.6 ± 1.3	55.9 ± 1.2	57.9 ± 2.4 º
Stem length (cm)	***	100.5 ± 14.5	139.8 ± 14.9 *	132.7 ± 12.9 *	131.4 ± 11.6 *	129.4 ± 24.9
Stem weight (g)	***	21.9 ± 6.3 *	29.3 ± 11.0 *	19.9 ± 8.6 *	9.9 ± 1.8	12.7 ± 4.8 *
Stem relative water content (%)		72.8 ± 8.3	59.2 ± 3.6	53.8 ± 1.8 º	47.9 ± 3.5	51.7 ± 1.9
Number of all leaves	*	52.4 ± 8.3	93.8 ± 7.1	76.4 ± 10.4 º	67.0 ± 8.2	62.0 ± 17.7
Weight of all leaves (g)	**	12.9 ± 3.9	18.2 ± 5.6	12.8 ± 3.9	11.4 ± 3.6	14.3 ± 8.6
Weight of a single leaf (g)	**	0.2 ± 0.05	0.2 ± 0.06	0.2 ± 0.04	0.2 ± 0.08	0.2 ± 0.12
Relative water content of all leaves (%)	*	35.2 ± 10.1 *	47.8 ± 16.3	55.1 ± 17.1	67.3 ± 34.9	62.5 ± 22.2
Number of assimilating, green, leaves		43.0 ± 8.9	70.3 ± 7.3	60.9 ± 2.4	60.1 ± 5.9 º	44.9 ± 16.1
Weight of assimilating, green, leaves (g)	**	11.9 ± 3.7	16.9 ± 5.6	11.9 ± 3.4	10.8 ± 3.4	12.4 ± 8.6
Weight of a single assimilating, green, leaf (g)	**	0.3 ± 0.1	0.2 ± 0.1	0.2 ± 0.1	0.2 ± 0.1	0.3 ± 0.1
Relative water content of assimilating, green, leaves (%)	**	75.1 ± 4.0	67.9 ± 3.1	64.9 ± 3.1	65.6 ± 0.8 ***	66.2 ± 4.5
Number of non-assimilating, brown, leaves	*	9.3 ± 0.8	23.5 ± 5.0 *	15.6 ± 8.0	6.9 ± 3.7	17.1 ± 10.6
Weight of non-assimilating, brown, leaves (g)	**	0.9 ± 0.4 *	1.3 ± 0.4 *	0.8 ± 0.5	0.6 ± 0.4	1.9 ± 0.9
Weight of single non-assimilating, brown, leaf (g)		0.1 ± 0.42	0.06 ± 0.01	0.05 ± 0.02	0.09 ± 0.03	0.1 ± 0.05
Relative water content of non-assimilating, brown, leaves (%)		59.8 ± 16.9	36.9 ± 8.8	37.3 ± 7.3	55.4 ± 12.9	50.2 ± 14.3
Inflorescence length (cm)		-	-	35.2 ± 7.9	36.1 ± 10.9	29.9 ± 7.2
Inflorescence weight (g)		-	-	11.1 + 4.4	22.6 ± 14.9	16.9 ± 7.9
Inflorescence relative water content (%)	*	-	-	65.5 + 3.1	68.8 ± 4.2 *	64.8 ± 3.3
Stem EO yield (mg/kg)	**	0.7 ± 0.8	6.2 ± 5.7	4.7 ± 4.0	12.3 ± 13.8 *	7.8 ± 4.6 **
Leaf EO yield (mg/kg)	*	1.9 ± 2.5	5.9 ± 6.2	3.5 ± 2.9 *	17.9 ± 10.7 **	248.3 ± 29.7 **
Inflorescence EO yield (mg/kg)		-	-	6.7 ± 9.3	20.9 ± 10.4	18.5 ± 6.7 *^,^º

**Table 2:** Selected morphological traits, essential oil yields (mg/kg) of the *Solidago canadensis* from medial invaded stands and significant mutual distinctions between the B versus C (°) categories of sampling stands, evaluated generally and for separated sampling terms, with three levels of significance (° *p* < 0.05; °° *p* < 0.01; °°° *p* < 0.001).

Morphological Traits		B Category 50–75% (Middle Degree of Invasion)				
		May/June	June/July	August	September	October
Plant height (cm)	°°	95.2 ± 8.9	121.8 ± 11.6	162.9 ± 6.8 °	146.9 ± 13.3	140.9 ± 22.4
Entire plant weight (g)		32.3 ± 5.9 °	42.0 ± 8.5	60.5 ± 13.8	59.1 ± 25.5	45.3 ± 9.3
Relative water content of entire plant (%)		73.2 ± 3.4	61.2 ± 2.6	56.9 ± 1.8	55.9 ± 6.5	49.6 ± 6.2 °
Stem length (cm)	°°°	95.2 ± 8.9	121.8 ± 11.6	127.7 ± 3.2 °	115.9 ± 10.1	110.4 ± 17.3
Stem weight (g)	°°°	19.5 ± 3.0 °	24.3 ± 5.5	18.9 ± 2.7 °°	10.1 + 2.3	11.9 ± 6.0
Stem relative water content (%)		72.8 ± 8.3	59.2 ± 3.6	53.8 ± 1.8 °	47.9 ± 3.5	51.7 ± 1.9
Number of all leaves	°°°	50.3 ± 8.4	89.5 ± 12.4	89.2 ± 7.3 °°	79.8 ± 6.6 °	68.4 ± 11.1
Weight of all leaves (g)	°°	13.1 ± 5.6 °	17.2 ± 2.9	15.5 ± 1.7 °°	11.5 ± 3.1	8.9 ± 4.1
Weight of a single leaf (g)		0.3 ± 0.01 °°	0.2 ± 0.02 °	0.2 ± 0.01	0.2 ± 0.04	0.1 ± 0.04
Relative water content of all leaves (%)		32.3 ± 5.9 °	42.0 ± 8.5	60.5 ± 13.8	59.1 ± 25.5	45.3 ± 9.3
Number of assimilating, green, leaves		44.0 ± 6.9	67.6 ± 8.8	69.3 ± 3.5 °°	68.1 ± 3.4 °	48.1 ± 23.9
Weight of assimilating, green, leaves (g)	°	12.4 ± 2.7 °	15.9 ± 2.9	13.9 ± 1.6 °°	10.5 ± 3.0	7.4 ± 4.9
Weight of a single assimilating, green, leaf (g)		0.3 ± 0.02 °°	0.2 ± 0.02 °	0.2 ± 0.02	0.2 ± 0.05	0.2 ± 0.04
Relative water content of assimilating, green, leaves (%)		75.4 ± 1.4	67.1 ± 1.7	63.1 ± 1.7	61.8 ± 3.4	64.2 ± 2.4
Number of non-assimilating, brown, leaves	°°	7.2 ± 0.5	21.9 ± 4.4 °	19.9 ± 9.3 °	11.8 ± 9.1	20.3 ± 13.9
Weight of non-assimilating, brown, leaves (g)	°	0.7 ± 0.3 °	1.3 ± 0.1 °°	1.6 ± 0.9 °	1.04 ± 0.7	1.5 ± 1.2
Weight of single non-assimilating, brown, leaf (g)		0.09 ± 0.03 °	0.06 ± 0.01 °	0.08 ± 0.03	0.10 ± 0.03	0.07 ± 0.01
Relative water content of non-assimilating, brown, leaves (%)		65.9 ± 8.7	42.5 ± 8.5	44.2 ± 11.4	49.5 ± 9.6	39.7 ± 1.3
Inflorescence length (cm)		-	-	35.2 ± 5.6	31.4 ± 7.8	30.6 ± 7.3
Inflorescence weight (g)		-	-	13.7 ± 8.3	18.5 ± 10.2	12.1 ± 2.9
Inflorescence relative water content (%)		-	-	66.5 ± 2.5	64.9 ± 2.5	62.4 ± 3.6
Stem EO yield (mg/kg)		1.6 ± 2.2	11.5 ± 26.1	4.7 ± 2.6	4.6 ± 3.8	8.8 ± 6.5 °°
Leaf EO yield (mg/kg)		4.4 ± 6.7	14.4 ± 31.7	11.7 ± 8.3	12.1 ± 10.5	7.2 ± 5.2
Inflorescence EO yield (mg/kg)		-	-	25.2 ± 31.4	15.5 ± 11.7	9.3 ± 7.8

**Table 3:** Selected morphological traits and essential oil yields (mg/kg) of the *Solidago canadensis* from mildly invaded stands.

Morphological Traits	C Category 25–50% (Mild Degree of Invasion)				
	May/June	June/July	August	September	October
Plant height (cm)	78.9 ± 16.1	93.7 ± 31.4	123.9 ± 31.4	129.7 ± 39.5	119.9 ± 35.3
Entire plant’s weight (g)	19.5 ± 7.1	22.7 ± 15.4	33.7 ± 22.7	52.3 ± 41.2	44.5 ± 26.7
Relative water content of entire plant (%)	70.9 ± 4.7	62.6 ± 2.8	57.6 ± 2.2	60.1 ± 7.9	59.1 ± 3.6
Stem length (cm)	78.9 ± 16.1	93.7 ± 31.4	95.8 ± 21.3	96.2 ± 27.5	93.5 ± 25.2
Stem weight (g)	11.0 ± 5.0	12.4 ± 9.3	9.2 ± 3.3	6.5 ± 4.3	5.2 ± 3.4
Stem relative water content (%)	68.8 ± 6.4	58.9 ± 2.9	53.2 ± 0.9	54.0 ± 16.3	51.2 ± 2.3
Number of all leaves	48.4 ± 3.4	70.1 ± 21.0	68.6 ± 6.8	63.6 ± 7.8	60.1 ± 10.9
Weight of all leaves (g)	8.3 ± 2.1	9.9 ± 5.9	7.9 ± 3.0	9.5 ± 3.0	10.7 ± 3.6
Weight of a single leaf (g)	0.2 ± 0.03	0.1 ± 0.03	0.1 ± 0.06	0.2 ± 0.07	0.2 ± 0.05
Relative water content of all leaves (%)	19.5 ± 7.1	22.7 ± 15.4	33.7 ± 22.7	52.3 ± 41.2	44.5 ± 26.7
Number of assimilating, *green*, leaves	41.7 ± 2.9	59.0 ± 13.5	61.9 ± 9.1	58.4 ± 5.9	46.7 ± 17.8
Weight of assimilating, *green*, leaves (g)	8.0 ± 2.1	9. 5 ± 5.5	7.7 ± 2.9	9.1 ± 3.0	9.2 ± 4.6
Weight of a single assimilating, *green*, leaf (g)	0.2 ± 0.04	0.2 ± 0.05	0.1 ± 0.07	0.2 ± 0.07	0.2 ± 0.05
Relative water content of assimilating, *green*, leaves (%)	73.8 ± 2.7	67.4 ± 3.4	61.3 ± 4.8	59.3 ± 1.5	52.8 ± 14.3
Number of non-assimilating, *brown*, leaves	6.6 ± 2.5	11.1 ± 7.6	6.7 ± 2.5	5.1 ± 4.6	13.4 ± 7.8
Weight of non-assimilating, *brown*, leaves (g)	0.3 ± 0.1	0.5 ± 3.8	0.3 ± 0.2	0.4 ± 0.4	4.5 ± 1.9
Weight of single non-assimilating, *brown*, leaf (g)	0.05 ± 0.01	0.05 ± 0.01	0.04 ± 0.01	0.07 ± 0.03	0.09 ± 0.07
Relative water content of non-assimilating, *brown*, leaves (%)	52.8 ± 11.2	50.9 ± 11.4	41.4 ± 10.4	43.9 ± 11.3	34.7 ± 11.9
Inflorescence length (cm)	-	-	28.1 ± 11.8	33.5 ± 15.7	26.4 ± 11.1
Inflorescence weight (g)	-	-	8.1 ± 8.0	17.9 ± 17.4	13.9 ± 11.4
Inflorescence relative water content (%)	-	-	67.3 ± 0.9	62.2 ± 2.9	51.6 ± 13.7
Stem EO yield (mg/kg)	1.7 ± 3.0	2.5 ± 3.2	4.3 ± 4.6	3.5 ± 2.0	3.3 ± 2.2
Leaf EO yield (mg/kg)	3.9 ± 3.6	3.6 ± 4.0	8.1 ± 5.5	6.0 ± 8.0	3.9 ± 3.1
Inflorescence EO yield (mg/kg)	-	-	12.1 ± 17.8	19.5 ± 28.4	8.4 ± 8.3

Single values of morphological traits represent means ± standard deviations (SD) from the 40 values (10 specimens × 4 sampling stands of the identical sampling stand category, according to degree of invasion A1–4, B5–8, C9–12), presented in the separate sampling terms. Single values of EO yields represent means ± standard deviations (SD) from the particular values (weight of the EO extracted from the 0.020 kg of the dry *Solidago canadensis* biomass) from 4 sampling stands of the identical sampling stand category according to the degree of invasion, presented for the separate sampling terms, sampling stand category and separated plant organs of *Solidago canadensis* used for the extraction. Values ± SD were determined using univariate statistic. Differences between the sampling stands categories in the selected morphological traits and EO yields were assessed using Two-way and One-way ANOVA, with three levels of significance (*p* < 0.05; *p* < 0.01; *p* < 0.001), where º indicates differences between the A versus B, * A versus C, and ° B versus C categories.

**Table 4:** Geographical coordinates and variables used for the Canadian goldenrod sampling stands characterization.

n.	Category	GPS		r.a. (%)	pH	W (%)	Area (ha)	int.
1	A	49°0′30.89″ N	21°20′6.69″ E	75–100	6.7	29.66	0.342	0
2	A	49°0′18.67″ N	21°16′13.28″ E	75–100	6.9	20.75	0.439	0
3	A	49°0′50.51″ N	21°12′43.90″ E	75–100	6.6	24.25	0.322	0
4	A	48°57′1.67″ N	21°21′19.30″ E	75–100	6.6	31.75	0.164	1
5	B	48°53′46.87″ N	21°17′55.67″ E	50–75	6.5	18.96	0.190	0
6	B	49°1′56.26″ N	21°15′13.30″ E	50–75	6.7	23.32	0.275	1
7	B	48°58′0.13″ N	21°17′2.22″ E	50–75	6.6	22.75	8.408	0
8	B	48°59′33.18″ N	21°12′38.96″ E	50–75	6.5	28.20	0.674	0
9	C	48°59′34.40″ N	21°12′53.02″ E	25–50	6.3	20,91	2.913	1
10	C	48°58′12.88″ N	21°18′58.71″ E	25–50	6.3	23.76	25.91	0
11	C	48°58′4.97″ N	21°19′25.90″ E	25–50	5.6	39.03	0.661	0
12	C	49°0′26.85″ N	21°20′13.25″ E	25–50	6.6	34.67	0.660	0

Abbreviation and notes: n.—number of sampling stand, category A = stands with the heavy degree of invasion, B = stands with the middle degree of invasion, and C = stands with the mild degree of *Solidago canadensis* invasion, GPs—geographical gps coordinates, r.a.—relative % abundance of the *Solidago canadensis* within the vegetation cover, pH—soil reaction, W—soil moisture (%), area—area of sampling stand (ha), int. —measure of interventions responded to the number of mowings per research period.

## Data Availability

The data presented in this study are available upon reasonable request from the corresponding author.
